# Novel transcatheter aortic valve replacement devices

**DOI:** 10.1016/j.xjse.2024.100040

**Published:** 2024-12-24

**Authors:** Ankur Sethi, Mark Russo

**Affiliations:** a Division of Cardiology, Department of Medicine, Robert Wood Johnson University Hospital, New Brunswick, NJ; b Division of Cardiothoracic Surgery, Department of Surgery, Rutgers Robert Wood Johnson Medical School, New Brunswick, NJ

**Keywords:** new TAVR, aortic regurgitation, future TAVR

**Figure:**
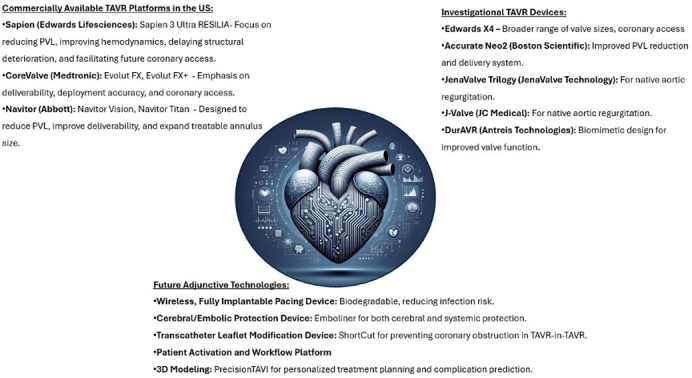
Advances and improvements in transcatheter aortic valve technology.

**Central Message**

TAVR has improved significantly since its inception. Innovations are now focused on coronary access, hemodynamics, durability, and feasibility of TAVR-in-TAVR.

**Perspective**

Transcatheter aortic valve replacement is now the most common form of aortic valve replacement in the United States. Understanding the features of different valve platforms is vital for optimal outcomes. We provide a comprehensive overview of the latest transcatheter aortic valve replacement platforms and adjunctive devices currently approved or under investigation in the United States.

Transcatheter aortic valve replacement (TAVR) has revolutionized the management of aortic stenosis (AS), offering a less-invasive alternative to surgical aortic valve replacement. Since its approval in the United States in 2011 for patients with severe AS at high or extreme surgical risk, TAVR has undergone continuous advancements. Early-generation TAVR devices prioritized reducing periprocedural complications like paravalvular leak, stroke, and major vascular complications. These complications have become increasingly uncommon due to iterative improvements in device design. Today, TAVR is an established treatment across the entire risk spectrum for patients with AS. Because significant progress has been made, the focus of technological innovation has shifted to addressing long-term issues. These include optimizing valve hemodynamics, mitigating structural valve deterioration, ensuring ease of coronary access and intervention, and enhancing the feasibility of future TAVR-in-TAVR procedures—collectively referred to as the lifetime management of patients with AS.

This article provides a comprehensive review of the latest TAVR platforms approved or under investigation in the United States. It highlights the design features that enhance the safety and efficacy of established transcatheter heart valves (THV) and explores emerging platforms and adjunctive technologies poised to shape the future of TAVR in the United States.

## Commercially Available TAVR Platforms in the United States

**Figure 1:**
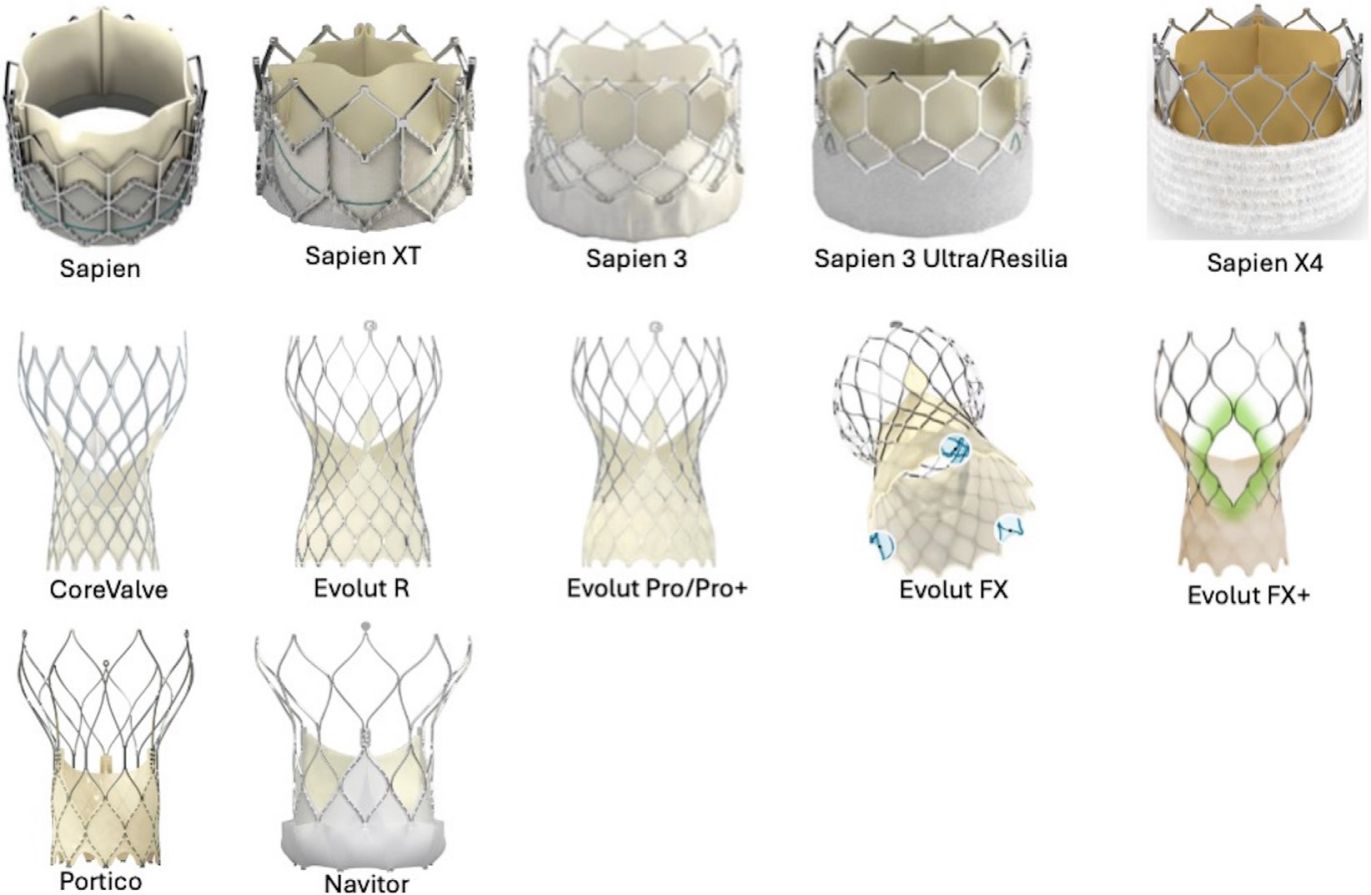
Commercially available transcatheter aortic valve platforms in the United States. Panel 1, Edwards Sapien platform and its iterations. Panel 2, Medtronic CoreValve platform and its iterations. Panel 3, Abbott Cardiovascular Portico platforms and its iterations. Old to new from left to right. Sapien X4 is the latest version of the Sapien platform is investigational and not yet Food and Drug Administration approved.

The rapidly expanding indications for TAVR underscore the need for devices capable of addressing diverse patient populations and complex anatomical variations. Currently, 3 valve platforms approved by the Food and Drug Administration (FDA) are available for commercial use in the United States for TAVR in patients with severe AS. These platforms, along with their iterations, aim to enhance valve delivery and deployment, improve coronary access, reduce the incidence of paravalvular leak and pacemaker implantation, and expand the range valve sizes. The design of these valves and their subsequent iterations are shown in Figure 1. The characteristics of commercially available and investigational aortic THV are shown in Table 1.

**Table 1:** Characteristics of transcatheter aortic valves commercially available or undergoing investigation in the US

Valve platform	Sapien (Edwards Lifesciences)	CoreValve/Evolut (Medtronic)	Navitor (Abbott)	Acurate (Boston Scientific)	JenaValve (JenaValve Technology)	J valve (JC Medical)
Frame type	Cobalt chromium	Nitinol	Nitinol	Nitinol	Nitinol	Nitinol
Frame expansion	Balloon-expandable	Self-expanding	Self-expanding	Self-expanding	Self-expanding	Self-expanding
Leaflets position	Intra-annular	Supra-annular	Intra-annular	Supra-annular	Supra-annular	Supra-annular
Leaflets tissue	Bovine	Porcine	Bovine	Porcine	Porcine	Bovine
Valve sizes	20, 23, 26, and 29 mm	23, 26, 29, and 34 mm	23, 25, 27, 29, and 35 mm	23, 25, and 27 mm	23, 25, and 27 mm	22, 25, 28, 31, and 34 mm
Labeled annulus use diameter	18.6-29.5 mm	18-30 mm	19-30 mm	21-27 mm	21-28.6 mm	18-33 mm
Sheath size	14Fr, 16Fr (29 mm valve)	14Fr, 16Fr (34 mm valve)	14Fr, 15Fr (>25 mm valves)	14Fr	18Fr (equivalent)	18Fr
Minimum vessel diameter required	5.5 mm, 6 mm (29 mm valve)	5 mm, 6 mm (34 mm valve)	5 mm, 5.5 mm (>25 mm valves)	5.5 mm	5.5 mm	5.5 mm
Valve lesion studied/being studied in the United States	Native aortic stenosis, aortic bioprosthesis failure	Native aortic stenosis, aortic bioprosthesis failure	Native aortic stenosis	Native aortic stenosis	Native aortic regurgitation	Native aortic regurgitation
US Food and Drug Administration approved	Yes (the latest iteration X4 is still investigational)	Yes	Yes (for high or extreme surgical risk patients only)	No	No	No

### Sapien

The Sapien 3 Ultra Resilia (Edwards Lifesciences) is the currently marketed fifth-generation iteration of the Sapien platform, launched during the last quarter of 2022. The Sapien valve system features a balloon-expandable cobalt-chromium frame with bovine pericardial leaflets and a polyethylene terephthalate skirt. Over the years, the valve system has undergone significant advancements, including improvements in frame height, outflow cell size and design, sheath size (reduced from 22Fr-24Fr to 14Fr-16Fr), sheath and dilator design, external skirts, and leaflet tissue treatment. The 5-year follow-up of the landmark the placement of aortic transcatheter valves 3 (PARTNER 3) trial demonstrated that among low-risk patients with severe, symptomatic AS, there was no significant difference in the primary outcome—a composite of death, stroke, or valve-related rehospitalization—between TAVR with Sapien 3 and surgical aortic valve replacement.^1^ Sapien 3 Ultra incorporates an outer sealing skirt that extends 40% higher above the inflow compared with Sapien 3, aiming to reduce the paravalvular leak. Building on these enhancements, Sapien 3 Ultra Resilia incorporates the Resilia anticalcification treatment, which includes a capping process and tissue preservation with glycerol instead of glutaraldehyde to eliminate exposure to free aldehyde in bovine tissue. This modification is aimed to delay structural valve deterioration. It also features a more robust outer skirt for the 29-mm valve to decrease paravalvular leak and revised leaflet suspension in 20- and 23-mm valves with the goal of improving valve durability and hemodynamic performance. In a propensity score-matched analysis, the Sapien 3 Ultra Resilia was associated with a lower mean gradient and larger effective aortic valve area compared with the Sapien 3/Sapien 3 Ultra. Additionally, the 29-mm valve size demonstrated reduction in paravalvular leaks, although there was no difference in death, stroke, and bleeding at 30-day follow-up.^2^

The most recent iteration of this platform, Sapien X4, is currently investigational and not commercially available. Sapien X4 introduces 3 significant enhancements over the Sapien 3 platform. It features 3 valves sizes that functionally provide 16 different deployment diameters in increments of 0.5 mm based on balloon inflation volume. Like the Sapien 3 Ultra, it includes Resilia tissue treatment, but with a lower frame height, larger outflow cells, and a central marker to assist in commissural alignment. These modifications are designed to facilitate future coronary access and valve-in-valve interventions. The safety and effectiveness of balloon-expandable bioprosthetic SAPIEN X4 transcatheter heart valve (ALLIANCE) and ALLIANCE AVIV single-arm studies are currently evaluating Sapien X4 in patients with symptomatic severe AS and failing bioprosthetic valve, respectively.^3^

### CoreValve

The Evolut FX (Medtronic) is the fourth-generation self-expanding valve of the CoreValve platform, featuring several enhancements. The CoreValve platform utilizes self-expanding nitinol frame and 3 single-layer supra-annular porcine pericardium leaflets (except for the first generation). Subsequent iterations—Evolut R and Evolut Pro—Introduced improvements including a shorter frame height, a lower profile inline delivery system for improved delivery (reduced from 18Fr-20Fr to 14Fr-18Fr), the ability to fully recapture the valve, and an external pericardial wrap to minimize paravalvular leak. Building on this platform, the Evolut FX includes 3 radio-opaque gold hat markers located 3 mm from the valve's inflow to assist with deployment depth and commissure alignment. The delivery system has now changed from double to single spline, coupled with a smoother nose cone to enhance flexibility and ease of delivery during femoral artery insertion. In addition to these enhancements, the most recent iteration, Evolut FX +, approved by FDA in March 2024, features larger diamond shape cells to facilitate coronary access. In first-in-human experience, 226 patients underwent transfemoral TAVR using Evolut FX valve system at 9 centers in the United States.^4^ optimal hat marker orientation in the cusp overlap or 3-cusp view on fluoroscopy and commissural alignment (defined as within 30% of native or prosthetic valve commissure) were achieved in 98.4% and 96.5% patients, respectively. Meanwhile, 30-day mortality, new pacemaker implantation, and greater than mild paravalvular leak were reported in 1.3%, 11.9%, and 0.9% of cases, respectively. These outcomes compare favorably to 30-days outcomes for earlier-generation valves (CoreValve, Evolut R, and Evolut Pro) in a contemporary US population with a mortality of 4.1%, 3%, and 2.7%; pacemaker implantation rate of 22.4%, 18%, and 16.9%; and more than mild aortic regurgitation in 8.3%, 5.4%, and 3.4% of patients.^5^

### Navitor Vision

The Navitor valve is a third-generation iteration of the Portico platform (Abbott) currently approved in the United States for patients with severe AS at high or extreme surgical risk. The Portico platform features nitinol frame with large-cell cylindrical nontapered design and intra-annular bovine pericardial leaflets. The Navitor valve introduces an outer and inner fabric cuff, termed NaviSeal, located around the inflow. This cuff is specifically designed to enhance apposition in calcified annuli to reduce paravalvular leak. This valve design along with FlexNav delivery system constitutes Navitor THV system. The newer FlexNav delivery system has a smaller delivery profile (14Fr-15Fr), hydrophilic coating, and stability layer to improve deliverability and precise valve deployment. In Portico NG (next generation), a multicenter global study, use of Navitor was associated with mortality, major vascular complications, and pacemaker implantation in 1.9%, 4.2%, and 19%, respectively, in high or extreme risk patients at 30-day follow-up.^6^

Recently, there have been 2 significant enhancements to the platform. First, an addition of radiopaque markers 3 mm from the inflow to aid in precise implantation depth, marketed as the Navitor Vision. Second, the platform has expanded the range of annuli that can be treated by offering the Navitor Titan for patients with large annuli (ie, a perimeter measuring between 85 and 95 mm).^7^ To expand indications to low and intermediate risk patients with symptomatic AS, the evaluation of the navitor transcatheter heart valve in low and intermediate risk patients who have severe, symptomatic, aortic stenosis requiring aortic valve replacement (ENVISION) a global multicenter trial, will randomize patients to either the Navitor THV or a commercially available THV evaluating a primary outcome of all-cause mortality or stroke at 12 months.^8^

## Investigational Devices

### Accurate Neo2

**Figure 2:**
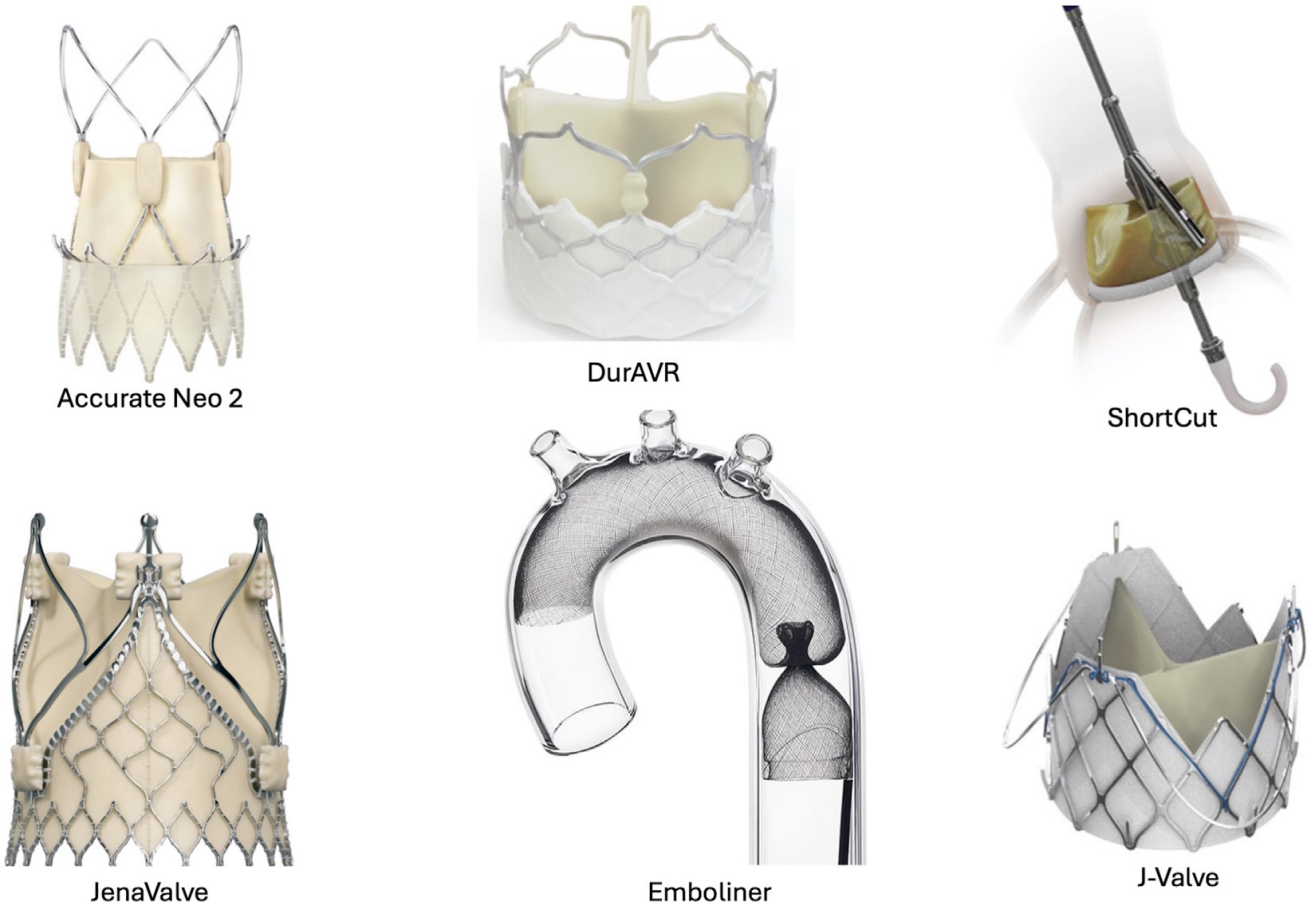
Current investigational transcatheter aortic valves and adjunctive devices in the United States.

As shown in Figure 2, The Acurate Neo (Boston Scientific) is a supra-annular THV featuring a self-expanding nitinol frame, porcine pericardial leaflets, and inner and outer skirt covering the inflow. It utilizes a unique top-to-bottom deployment mechanism that, along with protruding structure of the upper crown, is designed to enable stable deployment without the need for rapid pacing because it does not obstruct the left ventricle outflow. The upper crown provides supra-annular anchoring and caps the native leaflets to facilitate coronary clearance. It has lower radial force aimed at avoiding mechanical injury and the need for pacemaker implantation. safety and efficacy comparison of two TAVI systems in a prospective randomized evaluation (SCOPE) I, a randomized noninferiority trial, compared the Acurate Neo to the Sapien 3 valve in symptomatic patients with severe AS at increased surgical risk.^9^ However, Acurate Neo did not achieve noninferiority, primarily driven by higher acute kidney injury and paravalvular aortic regurgitation (moderate or greater). Additionally, a secondary analysis of the primary end point noted superiority of the Sapien 3 over the Acurate Neo. In SCOPE II, a randomized noninferiority trial, comparing the Acurate Neo to the Evolut R/Pro in symptomatic patients with severe AS at increased surgical risk,^10^ Acurate Neo again failed to meet noninferiority, and exhibited a higher cardiac mortality and moderate or more paravalvular aortic regurgitation but lower pacemaker rates.

The latest iteration, Acurate Neo2, introduces a 60% longer pericardial skirt to address the paravalvular leak, and features a lower profile atraumatic delivery system. The Acurate IDE is an international randomized trial that compared the Acurate Neo2 to the Sapien 3 and Evolut Pro or their commercially available iterations in 1500 patients with severe symptomatic AS.^11^ However, the Acurate Neo2 failed to meet noninferiority for a composite of death, stroke, and rehospitalization at 1 year. Limited physician experience and valve underexpansion were cited as potential factors contributing to its underperformance.

### JenaValve Trilogy TAVR System

The JenaValve Triology system (JenaValve Technology) is 1 of the 2 THV currently under evaluation for use in native aortic regurgitation in the United States.^12^ It features a self-expanding nitinol frame with leaflets and an external skirt made of porcine pericardial tissue. The valve is equipped with 3 radiopaque locators that are designed to clip the native aortic valve leaflets to provide anchoring and seal around the valve (Figure 2). It is currently available in 3 sizes: 23, 25, and 27 mm accommodating annular perimeters ranging from 66 to 85 mm (up to 90 mm). The valve is delivered through an 18Fr equivalent 85-cm sheath, which is advanced all the way to the sinotubular junction. Deployment involves careful positioning of the locators using aortography and/or transesophageal echocardiogram, followed by leaflet capture and valve anchoring. Once appropriate position, leaflet capture, and valve anchoring is confirmed the valve is released. The transcatheter aortic valve implantation in patients with high-risk symptomatic native aortic regurgitation (ALIGN-R) study, a single arm prospective multicenter study, evaluated 180 symptomatic patients with greater than moderate native aortic valve regurgitation from 20 sites in the United States undergoing TAVR with a JenaValve. Recently published results demonstrated mortality, stroke, and moderate or greater aortic regurgitation at 30 days in 2%, 2%, and <1% of cases, respectively. Technical success was achieved in 97% of patients,^13^ although a high pacemaker rate (24%) was observed. A future study, the Aortic Regurgitation Trial Investigating Surgery versus Trilogy is planned for low and intermediate risk patients with more than moderate AR comparing TAVR versus surgery in a randomized design.

### J-Valve

J-Valve (JC Medical) is another investigational THV being studied for native aortic regurgitation in the United States. It features a self-expanding nitinol frame, 3 bovine pericardial leaflets, and 3 nitinol anchor rings to secure the native leaflets (Figure 2). The frame includes sinus cutouts to facilitate coronary access. The transfemoral system requires an 18Fr sheath and is available in 5 valve sizes: 22, 25, 28, 31, and 34 mm, accommodating annuli with perimeters 57 to 104 mm. In a multicenter compassionate-use registry, 27 patients underwent J-valve implantation for native aortic regurgitation. The procedural success was 81%, whereas 1 death and 2 strokes at 30-day follow-up.^14^ The J-Valve TF early feasibility study is a single arm multicenter study that will enroll 15 to 25 symptomatic patients in the United States with severe native aortic regurgitation and high surgical risk.^15^

### DurAVR

The DurAVR (Antreis Technologies) is a first-in-class, balloon-expandable, valve system featuring a trileaflet design molded from a single piece of bovine pericardium. The tissue is treated with proprietary ADAPT technology to improve mechanical properties and valve durability. Valve has an outer polyethylene terephthalate skirt. This design aims to closely mimic the native aortic valve function and flow pattern compared with available bioprostheses. The frame has open cell design and radiopaque markers to assist in commissural alignment and coronary access. In a first-in-human study of 13 patients, there was no death, stroke, or major bleeding but 1 case each of major aortic regurgitation and pacemaker implantation at 1 year.^16^ Despite small mean baseline annular size, the effective orifice area and mean gradient on follow-up were very favorable, resulting in no patients with patient-prosthesis mismatch.

## Future Adjunctive Technologies

As TAVR technology continues to evolve, several adjunctive technologies are poised to further enhance its effectiveness (Figure 2).

### Cerebral/Embolic Protection Device

Stroke remains 1 of the most feared complications after a successful TAVR. The Sentinel Cerebral Protection System (Boston Scientific) uses filters deployed in the right brachiocephalic and left carotid arteries via right radial or brachial access. Despite its innovative design, clinical trials have failed to demonstrate a significant reduction in periprocedural stroke rates compared with controls.^17^ The Emboliner embolic protection device (Emboline Inc) is a cylindrical nitinol filter mesh that is deployed from ascending aorta across the arch vessel into the descending aorta from the contralateral transfemoral access.^18^ Its 150-micron pore size not only captures the embolic debris but removes it from the body, therefore offering cerebral and systemic embolic protection. The Protect the Head-to-Head is a randomized, open label, multicenter noninferiority study comparing Emboliner embolic protection device to the Sentinel device in patients undergoing transfemoral TAVR.^19^

### Transcatheter Leaflet Modification Device

Coronary obstruction remains a significant concern during valve-in-valve TAVR, particularly in younger, low-risk patients requiring lifetime management of AS. Snorkel stenting is not considered ideal due to uncertain long-term patency and potential complexities during future reinterventions. Bioprosthetic or Native Aortic Scallop Intentional Laceration to Prevent Iatrogenic Coronary Artery Obstruction is a technically demanding procedure involving guide catheters, microcatheters, snaring, and electrified coronary wires for leaflet laceration. Although effective, its complexity has limited widespread adoption.^20^ The ShortCut (Pi-Cardia) is a dedicated device designed for bioprosthetic valve leaflet splitting before valve-in-valve TAVR. It includes a splitting element for a controlled splitting of leaflet and a positioning arm to guide the procedure using fluoroscopy and transesophageal echocardiography and protect the splitting element. The ShortCut study was a nonrandomized multicenter single-arm trial to demonstrate the efficacy and safety of the device in patients undergoing valve-in-valve TAVR for failed bioprosthetic valve and at high risk of coronary obstruction.^21^ Sixty patients recruited from 22 clinical sites underwent leaflet splitting before TAVR. The freedom from coronary obstruction was 95%, and 98.3% of patients were free from the primary safety end point of mortality or stroke within 7 days of procedure. To simplify Basilica, Transmural Systems and the National Heart, Lung, and Blood Institute have developed purpose-built guidewires and catheters. The Transmural Electrosurgery Leaflet Traversal and Laceration Evaluation Basilica TAVR is a single-arm study currently recruiting patients with prosthetic or native aortic valve at high or prohibitive risk for surgery and deemed likely to experience coronary obstruction to undergo leaflet laceration with the device before TAVR.^22^

## Artificial Intelligence in TAVR

Artificial intelligence (AI) is becoming increasingly integral to TAVR technology. AI can optimize patient activation strategies and enhance preoperative planning through simulation, leading to better procedural outcomes. With the ability to process vast amounts of data, AI can personalize treatment plans and predict complications, ensuring that each patient receives the best possible care. The PrecisionTAVI (DASI Simulations) is a computer simulation modeling to predict valve frame deformation and patient-specific interactions based on the valve type and size.^23^ The input consists of 2-dimensional computerized tomography images from which a 3-dimensional model of patient's anatomy is generated. Its performance has been validated against post-TAVR computed tomography scans with an error rate ≤2 mm. It is FDA approved for patient-specific simulation of TAVR for preprocedural planning.

## Conclusions

The advancements in TAVR technology represent disruptive change in the treatment of aortic valve disease. Recent innovations have focused on improving device design, enhancing delivery systems, and ensuring the long-term durability of the valves. Emerging platforms like the JenaValve Trilogy, J-Valve, and DurAVR offer promising alternatives to traditional TAVR devices, whereas adjunctive technologies like embolic protection and leaflet modification devices and AI-driven platforms have the potential to further revolutionize patient care. As TAVR technology continues to evolve, it promises to become an increasingly reliable and versatile treatment option, thereby improving patient outcomes worldwide.

## Conflict of Interest Statement

The authors reported no conflicts of interest.

The *Journal* policy requires editors and reviewers to disclose conflicts of interest and to decline handling or reviewing manuscripts for which they may have a conflict of interest. The editors and reviewers of this article have no conflicts of interest.
